# The Effects of Two Polymorphisms on p21^cip1^ Function and Their Association with Alzheimer’s Disease in a Population of European Descent

**DOI:** 10.1371/journal.pone.0114050

**Published:** 2015-01-27

**Authors:** Sharon C. Yates, Amen Zafar, Erzsebet M. Rabai, James B. Foxall, Sheila Nagy, Karen E. Morrison, Carl Clarke, Margaret M. Esiri, Sharon Christie, A. David Smith, Zsuzsanna Nagy

**Affiliations:** 1 Neuropharmacology and Neurobiology, College of Medical and Dental Sciences, School of Clinical and Experimental Medicine, University of Birmingham, Birmingham, B15 2TT, United Kingdom; 2 Department of Neuropathology, University of Oxford, Level 1, John Radcliffe Hospital, Oxford, OX3 9DU, United Kingdom; 3 OPTIMA, University of Oxford, Level 4, John Radcliffe Hospital, Oxford, OX3 9DU, United Kingdom; 4 Department of Pharmacology, University of Oxford, Mansfield Road, Oxford, OX1 3QT, United Kingdom; University of S. Florida College of Medicine, UNITED STATES

## Abstract

With the exception of ApoE4, genome-wide association studies have failed to identify strong genetic risk factors for late-onset Alzheimer’s disease, despite strong evidence of heritability, suggesting that many low penetrance genes may be involved. Additionally, the nature of the identified genetic risk factors and their relation to disease pathology is also largely obscure. Previous studies have found that a cancer-associated variant of the cell cycle inhibitor gene p21^cip1^ is associated with increased risk of Alzheimer’s disease. The aim of this study was to confirm this association and to elucidate the effects of the variant on protein function and Alzheimer-type pathology. We examined the association of the p21^cip1^ variant with Alzheimer’s disease and Parkinson’s disease with dementia. The genotyping studies were performed on 719 participants of the Oxford Project to Investigate Memory and Ageing, 225 participants of a Parkinson’s disease DNA bank, and 477 participants of the Human Random Control collection available from the European Collection of Cell Cultures. The post mortem studies were carried out on 190 participants. In the in-vitro study, human embryonic kidney cells were transfected with either the common or rare p21^cip1^ variant; and cytometry was used to assess cell cycle kinetics, p21^cip1^ protein expression and sub-cellular localisation. The variant was associated with an increased risk of Alzheimer’s disease, and Parkinson’s disease with dementia, relative to age matched controls. Furthermore, the variant was associated with an earlier age of onset of Alzheimer’s disease, and a more severe phenotype, with a primary influence on the accumulation of tangle pathology. In the in-vitro study, we found that the SNPs reduced the cell cycle inhibitory and anti-apoptotic activity of p21^cip1^. The results suggest that the cancer-associated variant of p21^cip1^ may contribute to the loss of cell cycle control in neurons that may lead to Alzheimer-type neurodegeneration.

## Introduction

Late-onset Alzheimer’s disease (AD) is a neurodegenerative disorder of the elderly that manifests in a myriad of symptoms including memory loss, confusion, and a reduced capacity for learning [1]. Whilst the heritability of AD is up to 80% [2], so far, only one genetic risk factor, the E4 variant of the Apolipoprotein E gene (ApoE), is consistently shown to be associated with an increased risk and reduced age of onset. Genome-wide association studies (GWAS) have identified additional genetic variants, on genes involved in a variety of pathways, which may lead to a small increase in AD risk [3–15]; however the individual contribution of each variant is small. It is likely that the variants work in combination to increase the risk of AD; and that the exact complement of variants differs from patient to patient.

One possible genetic risk factor for AD is a variant of p21^cip1^, a cyclin-dependent kinase inhibitor (CDKI) gene, which is already established to be associated with an increased risk of some cancers [16–22]. In a preliminary study, the variant (present in ~7% of populations of European descent according to the Ensembl genome database) was found to be associated with an increased risk and reduced age of onset of AD [23]. Furthermore, a number of GWAS [6,24–26] identify the 6p21 loci, on which the p21^cip1^ gene is located, as associated with AD risk: compatible with the hypothesis that the p21^cip1^ variant is a risk factor for AD development [23].

The G1/S checkpoint is partly regulated by the CDKIs: p21^cip1^, p27^kip1^ and p57^kip2^. P21^cip1^ inhibits the activity of cyclin/CDK4 and cyclin/CDK2 complexes, which are required for cell cycle progression through the G1 phase and G1/S checkpoint respectively. It suppresses E2F-dependent transcription of cell cycle proteins by binding to E2F transcription factor 1 (E2F1) [27]. It also inhibits the activity of proliferating cell nuclear antigen (PCNA), a protein required for DNA replication and repair. In addition, p21^cip1^ has been shown to regulate the G2/M checkpoint when expressed independently of p53 [28–31]. P21^cip1^ has an anti-apoptotic role by inhibiting the stress activated protein kinase (SAPK) and apoptosis signal-regulating kinase 1 (ASK1), as well as inhibiting Fas-mediated apoptosis [32]. A number of additional proteins interact with p21^cip1^ to alter its activity. For example, WISp39 has been shown to stabilise the p21^cip1^ protein [33]; whilst tumour susceptibility gene 101 (TSG101) enhances the cyclin/CDK inhibitory activity of p21^cip1^ by stabilising the p21^cip1^/cyclin/CDK complexes [33,34].

The association of the rare p21^cip1^ variant with cancer suggests that the variant has reduced function or expression: although this has not been experimentally verified. The genetic variant has substitutions at two DNA bases [19,21]: a cytosine to adenine substitution that induces a serine to arginine change at codon 31 of p21^cip1^ (p21 C98A, dbSNP rs1801270); and a cytosine to thymine change in the 3′-untranslated region (UTR) (p21 C70T, dbSNP rs1059234). The single nucleotide polymorphisms (SNPs) are only associated with cancer when they occur together [19,21]. The SNP at codon 31 of p21^cip1^ induces an amino acid change in the zinc finger domain [20,35] that lies between a cyclin (amino acids: aa 17–24) and CDK binding sites (aa 53–58) [33]. The SNP is located within the binding domains of TSG101 (aa 1–86), WISp39 (aa 28–56), E2F1 (aa 1–90) and the pro-apoptotic proteins: procaspase 3 (aa 1–33), SAPK (aa 1–84) and ASK1 (aa 1–140). It is not within the binding domain of the cyclins (aa 17–24 and 155–157), CDKs (aa 53–58 and 74–79), PCNA (aa 143–160), or various other proteins (reviewed in [33]). It is plausible that the SNP may reduce the cell cycle inhibitory activity of p21^cip1^ by reducing the strength of binding of p21^cip1^ to TSG101 and/or E2F1; and may reduce the anti-apoptotic activity of p21^cip1^ by reducing the strength of binding to the pro-apoptotic proteins. The SNP may also reduce the stability of the p21^cip1^ protein by interfering with WISp39 binding. Whilst the p21^cip1^ protein is intrinsically unstructured [36], the serine (polar uncharged) to arginine (positively charged) change induced by the SNP may alter the preference of the p21^cip1^ protein for certain tertiary structures, potentially altering its interaction with any of its protein targets. The SNP in the 3′-UTR of p21^cip1^ may alter expression, as this region of the mRNA interacts with various non-coding RNAs and proteins that regulate mRNA stability and translation efficiency [32].

The reported association of the p21^cip1^ variant with AD [23] is not surprising when interpreted in light of the cell cycle theory of AD, which postulates that neurons degenerate secondary to aberrant cell cycle activity [37–39]; and specifically loss of G1/S checkpoint control [23]. To investigate this further, we examine the association of the p21^cip1^ SNPs with AD and with Parkinson’s disease (PD) with dementia. We also investigate the association of the p21^cip1^ SNPs with various phenotypic features of a large AD cohort, including the accumulation of AD-related pathology in the brain. Furthermore, we investigate the effect of the SNPs on the function and expression of p21^cip1^ in vitro, by transiently transfecting human embryonic kidney cells (HEK-293) with either the common or rare p21^cip1^ variant.

## Methods and Materials

### Ethics Statement

Full ethical approval and written informed consent were obtained for all studies (NHS Research Ethics Committee approvals: 09/H0107/9, 07/Q2707/98, MREC/00/7/56a).

### Subjects

The genotyping studies were performed on 719 participants of the Oxford Project to Investigate Memory and Ageing (OPTIMA), 225 participants of PD GEN (a Parkinson’s disease DNA bank) and 477 participants of the Human Random Control (HRC) collections available from the European Collection of Cell Cultures (ECACC) (HRC plates 1–5). The OPTIMA, PD GEN and ECACC donors were all of European descent. The clinical diagnoses of the participants of OPTIMA and PD GEN are summarised in Table 1. Post-mortem brain tissue was available from 190 participants of the OPTIMA cohort through the Thomas Willis Oxford Brain Collection.

The clinical information provided by the OPTIMA team included: age of onset of dementia; age at death; and the results of annual cognitive performance tests (CAMCOG) [40]. Clinical diagnosis of AD was made using the NINCDS-ARDRA criteria [41]. The diagnosis of PD was made using the UK Parkinson’s Disease Society Brain Bank criteria [42]. Dementia in PD patients (PDGEN cohort) was established based on the presence of progressive deterioration in intellectual abilities that interferes with activities of daily living [43]. The post mortem brain tissues were characterised in detail by a clinical neuropathologist. The information available included the pathological diagnosis (CERAD protocol) [44] and the severity of AD as defined by Braak [45]. Subjects where pathologies other than AD were regarded as severe enough to contribute to the clinical dementia syndrome were excluded from the clinico-pathological analyses [46–48]. Demographic data on these patient groups is in S1 Table and S2 Table.

**Table 1:** The total number of subjects in each diagnostic category.

	**Control**	**AD**	**OD**	**AD/PD**	**MCI**	**PD**	**PD D**	**TOTAL**
**OPTIMA**	242	292	84	20	65	4	12	719
**PD GEN**	0	0	0	0	0	112	113	225
**TOTAL**	242	292	84	20	65	116	125	944

(AD: Alzheimer’s disease; OD: Other dementia; AD/PD: Alzheimer’s disease with Parkinson’s disease; MCI: Mild cognitive impairment; PD: Parkinson’s disease; PD D: Parkinson’s disease with dementia; OPTIMA: Oxford Project to Investigate Memory and Ageing; PD GEN: Parkinson’s disease DNA bank).

### Sample Preparation

RNA, DNA and protein were isolated from the lateral temporal, frontal and occipital lobe of each post-mortem subject by standard TRI-reagent protocol (Sigma). RNA was converted to cDNA with the High Capacity cDNA Reverse Transcription kit (Applied Biosystems) according to the manufacturer’s recommendation. Protein was stored in radio-imunoprecipitation buffer (0.1M NaCl, 0.01M TrisHCl, 1:500 EDTA, 400μg/ml phenylmethanesulfonylfluoride, 2μg/ml aprotinin and 1% sodium dodecyl sulphate).

### p21^cip1^ Genotyping

The SNPs at codon 31 (RS1801270) and in the 3′-UTR of p21^cip1^ (RS1059234) were genotyped as described in [19], using restriction enzymes Bsma1 and PstI (Biolabs) as recommended by the manufacturer.

### ELISA

Five markers of AD-related pathology were quantified by ELISA in the temporal, frontal and occipital lobe of each subject (Table 2) [49–52]. A protein sample with a high quantity of the protein-of-interest was selected for each standard curve; and samples analysed in triplicate. For each ELISA, 100 μl of protein, diluted in 0.1M Carbonate buffer (pH 9.6) to the optimal loading concentration (see Table 3), was loaded per well and incubated overnight at 4°C. Following blocking with 1% bovine serum albumin (Sigma) in PBS (Sigma), the samples were incubated with primary antibody at 4°C overnight, with secondary antibody at room temperature (RT) for 2 hours and with Streptavidin-HRP (1:200, R&D Systems) at RT for 2 hours. For antibody concentrations see Table 3. The reactions were visualised with o-phenylenediamine dihydrochloride (SIGMAFAST OPD); and reaction stopped with 25μl of 4M H_2_SO_4_. The calibration curve, obtained from the serial dilution of the standard curve sample, was used to estimate concentration of the protein based on their optical density readings.

**Table 2:** Markers of AD-related pathology that were quantified by ELISA in the brain.

**Marker**	**Description**
Beta-amyloid	39–43 amino acid cleavage product of APP that accumulates in the brain in AD.
Phospho-tau (p-tau)	Hyperphoshorylated (Ser 202/Thr 205) version of tau found in neurofibrillary tangles.
Neurofibrillary tangle (NFT) (epitope DC11)	A protein aggregate that accumulates in neurons in AD. DC11 is a conformational epitope of the neurofibrillary tangle that is highly AD specific.
Synaptophysin	38kDa glycoprotein located on neuronal synapses. A marker of synaptic density.
Growth associated protein 43 (GAP-43)	Neuron specific protein expressed on axonal growth cones during synaptic remodelling. A marker of synaptic remodelling activity.

**Table 3:** The protein loading concentration and antibody information corresponding to each ELISA.

**Protein**	**Loading conc. (μg/ml)**	**Primary antibody**	**Secondary antibody**
Beta-amyloid	2	Mouse monoclonal to amyloid-β (1:5000, Dako)	Biotinylated polyclonal rabbit anti-mouse IgG (1:2000, Dako)
p-tau	2.5	Mouse monoclonal to AT8 (1:1000, Innogenetics)	Biotinylated polyclonal rabbit anti-mouse IgG (1:2000, Dako)
DC11	4	Mouse monoclonal to DCII (1:500, Sigma)	Biotinylated polyclonal rabbit anti-mouse IgG (1:2000, Dako)
GAP-43	2	Rabbit polyclonal to GAP43 (1:1000, Chemicon)	Biotinylated polyclonal swine anti-rabbit IgG (1:2000, Dako)
Synapto-physin	2	Mouse monoclonal to synaptophysin (1:1000, Dako)	Biotinylated polyclonal rabbit anti-mouse IgG (1:2000, Dako)
p21^cip1^	4	Rabbit polyclonal to p21^cip1^ (1:500, ABcam)	Biotinylated polyclonal swine anti-rabbit IgG (1:2000, Dako)

### Q-PCR

Q-PCR was used to quantify p21^cip1^ mRNA in the frontal lobe of each subject, with normalisation to beta-actin content. Q-PCR systems were designed with the Universal Probe Library Design Centre (Roche Diagnostic); and consisted of primers: 5′-TGGGTGGTACCCTCTGGA-3′and 5′-TGAATTTCATAACCGCCTGTG-3′ with Universal probe 12 for p21^cip1^; and primers: 5′-TCAGCTGTGGGGTCCTGT-3′ and 5′-GAAGGGGACAGGCA GTGAG-3′ with Universal probe 24 for beta-actin. Each 20μl Q-PCR reaction consisted of 2μl neat cDNA, 2.5μM of each primer, 0.25μM Universal probe (Roche) and 2x Absolute Q-PCR mix (Thermo Scientific). Products were amplified with an initial 15 minute step at 95°C, followed by 50 cycles of 96°C for 15 sec, 62°C (beta-actin) or 58°C (p21^cip1^) for 30 sec, and 72°C for 30 sec.

### Cell Culture and Transfection

The prolonged consistent overexpression of p21^cip1^ leads to a complete cessation of cell division [53] and would prevent the detection of subtle effects on cell cycle regulation or apoptosis. Therefore we have chosen transient transfection in human embryonic kidney cells (HEK-293, Invitrogen) to study the effects of the p21^cip1^ variants on cell cycle, apoptosis and cell differentiation. The HEK-293 cells are easy to grow and easy to transfect. The cell line is capable of generating functional, mature proteins and is widely used for the biochemical/cell biological evaluation of expressed proteins in concert with functional analyses to establish pharmacological effects [54].

The cells were cultured in Dulbecco’s Modified Eagle’s Medium (DMEM) supplemented with 4500 mg glucose/litre (Sigma), 10% foetal calf serum (FCS) gold (PAA), and 2mM L-glutamine (Gibco), until 90–95% confluence. Cells were transfected with plasmids (ready to transfect TrueClones from Origene, in pCMV6-XL5 and pCMV6-XL4) designed to express either the common p21^cip1^ variant (IMAGE ID 2821049), the rare p21^cip1^ variant (with SNP at codon 31 and in the 3′-UTR; IMAGE ID 2822909), or no p21^cip1^ (empty vector negative control, EV NC, pCMV6-XL5) with Lipofectamine-2000 (Invitrogen). The presence/absence of the two polymorphisms in the purchased clones was confirmed by sequencing (Invitrogen service). Before transfection the cells were grown in full medium to reach 90–95% confluence (approximately 2 days). In preparation for transfection, 30μg of plasmid DNA was diluted in 1.875ml of Opti-MEM reduced serum medium (Gibco); and 75μl of Lipofectamine 2000 (Invitrogen) diluted in 1.875ml Opti-MEM. After 5 minute incubation, the two mixtures were combined and incubated at RT for 20 minutes. The 3.75ml mixture was added to a flask of cultured cells and mixed by gentle rocking. After 36 hours incubation the cells were dislodged with 1mM EDTA and collected for RNA and protein extraction or seeded into 96 well plates for cytometry. Transfection efficiency was calculated using the assumption that p21^cip1^ transfected cells stop proliferating while the non-transfected cells proliferate at their original proliferation rate of ~1 population doubling in 36 hours [55].

Following an overnight incubation the cells in the 96 well plates were fixed with Glyofixx for p21^cip1^ and beta-actin immunolabelling. The fixed cells were sequentially incubated with blocking solution (5% BSA and 0.1% triton-X in PBS), primary and secondary antibody, and propidium iodide (PI, 10μg/ml) supplemented with RNaseA (100μg/ml), prior to immunofluorescence, and combined image analysis and cytometry, with the Acumen Explorer Cytometer (TTP Labtech, Acumen Explorer Software version 3.1.12.). The antibodies were rabbit polyclonal to p21^cip1^ (ABcam, 1:500), mouse monoclonal to beta-actin (ABcam, 1:500), anti-rabbit IgG FITC (ABcam, 1:200) and anti-mouse IgG FITC (ABcam, 1:200).

**Figure 1:**
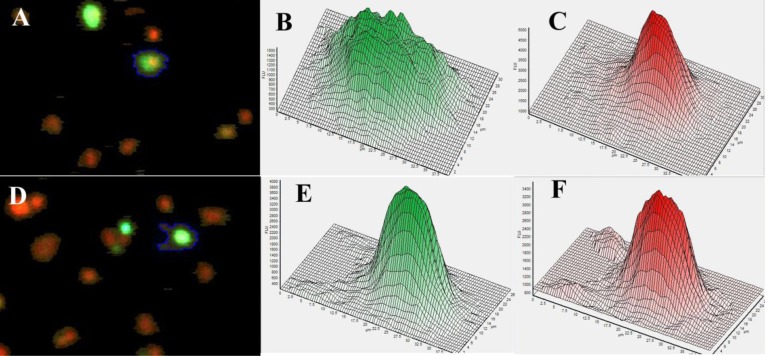
HEK293 cells transfected with p21cip1. Image of cells acquired from the Acumen cytometer. The p21^cip1^ was labelled by immunohistochemistry (green fluorescence). The DNA is labelled red by the PI. **Panel A:** The cell with the blue edge is an example of what we would accept as cytoplasmic protein expression. **Panel B** is the green fluorescence intensity histogram (as measured by the cytometer) over the surface of the same cell (with the blue edge in panel A) indicating a relatively uniform protein distribution in the cytoplasm around the nucleus. **Panel C** is the distribution histogram of the red fluorescence (DNA) from the same cell (with the blue edge in panel A). The distribution of the DNA is characteristic for a single cell. **Panel D**: The cell with the blue edge is an example of what we would accept as nuclear protein expression. **Panel E** is the green fluorescence intensity histogram (protein content as measured by the cytometer) over the surface of the same cell (with the blue edge in panel D). **Panel F** is the red fluorescence (DNA) from the same cell (with the blue edge in panel D). The overlap and identical distribution patterns of the protein (green label) and DNA (red label) is characteristic for nuclear proteins.

The PI stained cultures provided measurements of DNA content and nucleus size, and were analysed by standard cytometry methods [56] to determine cell cycle phase of the individual cells in the sample. The immunolabelled cells provided measurements of p21^cip1^ and beta-actin protein content per cell, and per nucleus, and allowed the categorisation of cells based on expression (positive/ negative) and subcellular localisation (Fig. 1). Single cells were defined by the size of the cell and shape of the PI distribution in the nucleus (as seen in fig. 1 panes C and F). The DNA histograms were gated based on published methods [57]. The gates were set separately for each 96 well plate to eliminate the effect of slight changes in excitation. The percentage of cells in G1, S and G2/M phases of the cell cycle were calculated as a fraction of the euploid cell population. Cells with sub-G1 DNA content were categorised as apoptotic while single cells with above-G2 DNA content were regarded as polyploid. The percentage of apoptotic cells was calculated as a fraction of the single cells. Clumps of multiple cells (identified by multiple nuclei within the same ‘object’) and debris (no DNA trace specific for nuclei as shown in Fig. 1C) were excluded from the analysis. Cytometric analysis of the cell cycle phases and apoptosis was carried out separately for p21^cip1^ positive and negative cells (as defined above) within each population. Analysis was carried out from 8 technical replicates of more than 5000 cells each at the time. The transfections were repeated on three separate occasions. The extracted RNA was converted to cDNA as described and used for p21^cip1^ Q-PCR analysis.

### Statistical Analysis

Data was analysed with the MedCalc statistical package, version 13.0.2.0. Departure from the Hardy-Weinberg equilibrium was tested using the Chi-square test. Odds ratios (OD) with 95% confidence intervals (CI) were used to determine the association of the p21^cip1^ variant with AD and with PD with dementia, relative to age-matched controls. In the post-mortem study, the D’Agostino-Pearson normality test was used to assess the distribution of datasets prior to statistical analysis and the most appropriate test selected in each case (for data that followed a normal distribution: ANOVA or MANOVA; for data that did not follow a normal distribution: Kruskal-Wallis). To eliminate the need for subgroups defined by the severity of AD, Z-scores were calculated for the pathology data taking into account the disease severity as defined by Braak [45]. The Z-scores were analysed by ANOVA.

Previous experiments with the same cell line indicate that even a 3 fold increase in the expression of the cyclin dependent kinase-like 3 (cdkl3, which drives the transition from G1 to S phase to increase cell proliferation) leads to only modest changes in proliferation rate (19% change) and cell cycle (11% change in the G1 population) [55]. Therefore any differences between the effect of the two p21^cip1^ variants, even if significant, may be modest and could be easily masked by the fast growing non-transfected cells. Additionally transient transfection is a stressful procedure, with relatively large experimental variability and cell cycle kinetics is greatly influenced by cell density in cultures. Therefore changes induced by the p21^cip1^ variants (common and rare) were analysed by comparing the cell populations successfully transfected with p21^cip1^ to both the empty vector negative control cells (EV NC) and to the non-transfected cell population within the same well. Differences between means larger than 2 standard deviations were accepted as statistically significant.

## Results

The rare p21^cip1^ haplotype had alterations at two polymorphic sites: at codon 31 (a cytosine to adenine substitution) and in the 3′-UTR (a cytosine to thymine substitution). Subjects that had one or two copies of the rare haplotype (with both substitutions) were classed as having variant p21^cip1^, whereas subjects that did not have a copy of the rare haplotype were classed as having common p21^cip1^.

**Figure 2:**
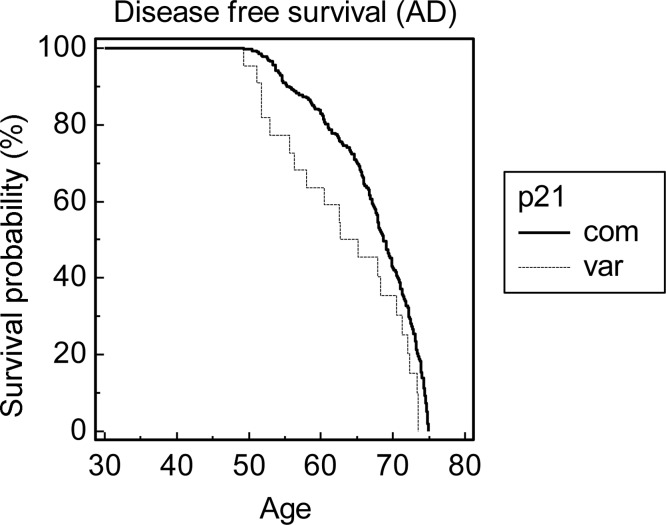
Kaplan-Meier probability distribution of disease free survival prior to the age of 75. The disease in question was dementia associated with AD. The graph shows the disease free survival probability of subjects prior to the age of 75, in subgroups defined by the p21^cip1^ genotype. Prior to the age of 75, the variant p21^cip1^ was associated with a significant reduction in the disease free survival compared to the common p21^cip1^ (hazard ratio: 1.698, p-value: 0.017). The x-axis represents the age in years. The y-axis represents the survival probability expressed as a percentage. The solid black line represents subjects that were homozygous for the common p21^cip1^. The broken grey line represents subjects that were heterozygous or homozygous for the variant p21^cip1^.

**Figure 3:**
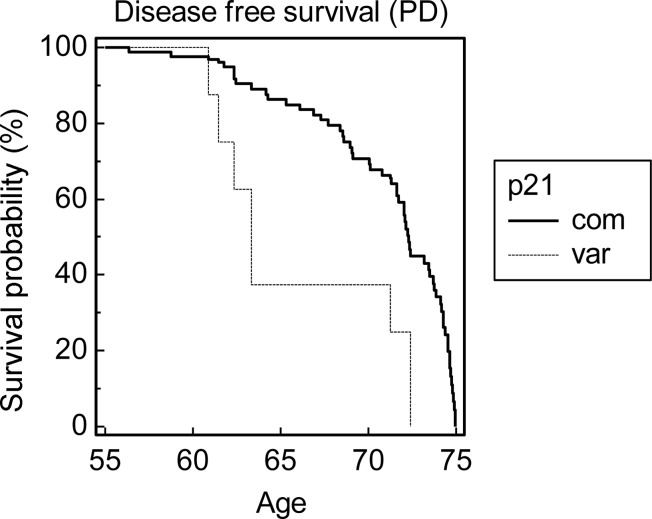
Kaplan-Meier probability distribution of disease free survival prior to the age of 75. The disease in question was dementia in Parkinson’s disease. The graph shows the disease free survival probability of subjects prior to the age of 75, in subgroups defined by the p21^cip1^ genotype. Prior to the age of 75, the variant p21^cip1^ was significantly associated with a reduction in the disease free survival compared to the common p21^cip1^ (hazard ratio: 3.239, p-value < 0.001). The x-axis represents the age in years. The y-axis represents the survival probability expressed as a percentage. The solid black line represents subjects that were homozygous for the common p21^cip1^. The broken grey line represents subjects that were heterozygous or homozygous for the variant p21^cip1^.

Of the 477 ECACC Human Random Control subjects that were genotyped, 435 were homozygous for the common variant of p21^cip1^, 39 were heterozygous, and 3 were homozygous for the rare variant. Of the 944 live and post-mortem subjects in the OPTIMA and PD GEN cohorts, 852 were homozygous for the common variant, 89 were heterozygous, and 3 were homozygous for the rare variant. The allele frequency of the rare variant in the ECACC Human Random Control cohort, and in the combined OPTIMA and PDGEN cohort, was 0.047 and 0.053 respectively (Table 4). The distribution of the rare p21^cip1^ haplotype followed the Hardy-Weinberg equilibrium in all the investigated cohorts. The presence of the variant was significantly associated with an increased risk of AD (odds ratio 1.796, p = 0.030, 95% CI: 1.057 to 3.051) and with PD with dementia (odds ratio 2.052, p = 0.022, 95% CI: 1.108–3.798), relative to age-matched controls (Table 5). There was a trend for an association between the rare variant and PD with dementia, relative to PD without dementia (age matched subjects), but the result failed to reach statistical significance (Table 5). Prior to the age of 75, the variant was associated with a reduced disease (dementia) free survival in relation to AD (hazard ratio 1.698, p = 0.017) (Fig. 2) and PD with dementia (hazard ratio 3.239, p < 0.001) (Fig. 3). Neither association was present above the age of 75 (data not shown). The sample size was not big enough to analyse a possible synergy between the p21^cip1^ haplotype and the ApoE genotype.

**Table 4:** The allele frequency of the p21^cip1^ variant in the various study groups.

**Subgroup**	**Collection**	**Variant P21^cip1^ allele count**	**Common P21^cip1^ allelecount**	**Variant allele frequency**
All subjects	ECACC HRC	45	909	0.047
All subjects	OPTIMA and PD GEN	95	1793	0.053
Controls and non-AD dementia	OPTIMA	23	637	0.035
AD	OPTIMA	38	586	0.061
PD without dementia	OPTIMA and PD GEN	11	213	0.049
PD with dementia	OPTIMA and PD GEN	20	270	0.069

For the OPTIMA and PD GEN collection, the variant allele frequency was analysed for the cohort as a whole, and in separate groups defined by diagnosis (diagnostic criteria outlined in the methods). AD refers to Alzheimer’s disease; PD to Parkinson’s disease; OPTIMA to the Oxford Project to Investigate Memory and Ageing; PD GEN to the Parkinson’s disease DNA Bank; and ECACC HRC to the European Collection of Cell Cultures Human Random Control.

**Table 5:** Odds ratios for disease in relation to the rare p21^cip1^ variant.

**DIAGNOSIS**	**Odds ratio (variant relative to common)**	**P-value**	**95% CI**
AD (versus controls and non-AD dementia)	1.796	< 0.05	1.057–3.051
PD with dementia (versus controls and non-AD dementia)	2.052	< 0.05	1.108–3.798
PD with dementia (versus PD without dementia)	1.433	Not significant	

The OPTIMA and PD Gen cohorts were separated into groups defined by diagnosis based on the diagnostic criteria outlined in the methods. Odds ratios in relation to the p21^cip1^ variant were calculated for the disease groups compared to age-matched controls.

### Post-Mortem Pathology Study

In the post-mortem study, the severity of AD was defined according to the Braak staging system[45]: with entorhinal, limbic and neocortical stage subjects in a preclinical, mild and advanced stage of AD respectively. The distribution of the ApoE genotypes in subgroups defined by the AD severity was similar to that of published datasets [58]. There was no significant difference in the distribution of the ApoE genotypes, or in the frequency of the ApoE4 allele, in the common p21^cip1^ group compared to the variant p21^cip1^ group, irrespective of the severity of AD (data not shown).

**Figure 4:**
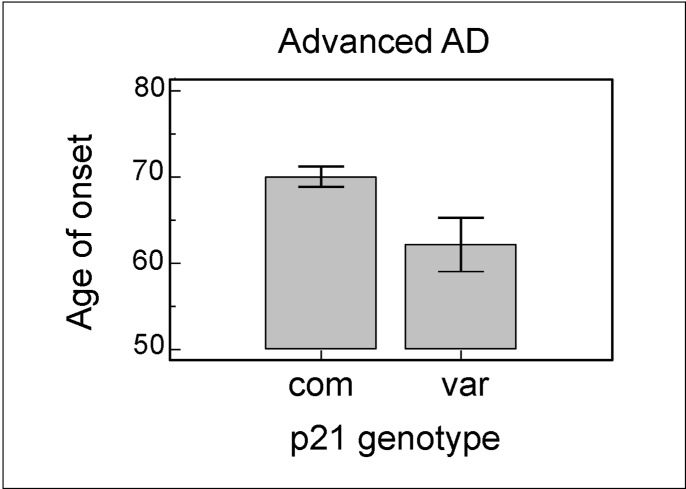
The effect of the p21^cip1^ genotype on the age of onset of AD. Of the subjects with advanced AD at post-mortem, the subjects with the variant p21^cip1^ had a significantly lower age of onset than subjects with the common p21^cip1^ (p-value: 0.016). The x-axis represents the p21^cip1^ genotype, with com and var representing subjects with the common and variant p21^cip1^ respectively. The y-axis represents the age at onset of AD in years. The top of the bars represent the mean. The error bars represent the standard error of the mean (SEM). Statistical test: one-way ANOVA.

There was a trend for the p21^cip1^ variant to be more common in subjects with advanced AD (allele frequency: 0.081) compared to subjects with pre-clinical (allele frequency: 0.052) and mild AD (allele frequency: 0.053) (Table 6). However, as we had a relatively small sample size, the result (odds ratio: 1.587) failed to reach statistical significance. The p21^cip1^ genotype had no effect on the age at death, the duration of AD, or the cognitive performance of subjects just prior to death, irrespective of AD severity (data not shown). However, of the subjects with advanced AD at post-mortem, the subjects with variant p21^cip1^ had an earlier age of onset of AD than subjects with common p21^cip1^ (p = 0.016) (Fig. 4). This effect on the age of onset was not present in the subjects in the earlier stages of AD.

**Table 6:** The allele frequency of the p21cip1 variant in groups defined by the severity of AD.

**Diagnosis**	**Variant allele count**	**Common allele count**	**Variant allele frequency**
**Pre-clinical**	4	72	0.052
**Mild AD**	7	125	0.053
**Advanced AD**	14	158	0.081

The diagnosis was defined according to the Braak staging system: with entorhinal, limbic and neocortical stage subjects in a pre-clinical, mild and advanced stage of AD respectively.

**Figure 5:**
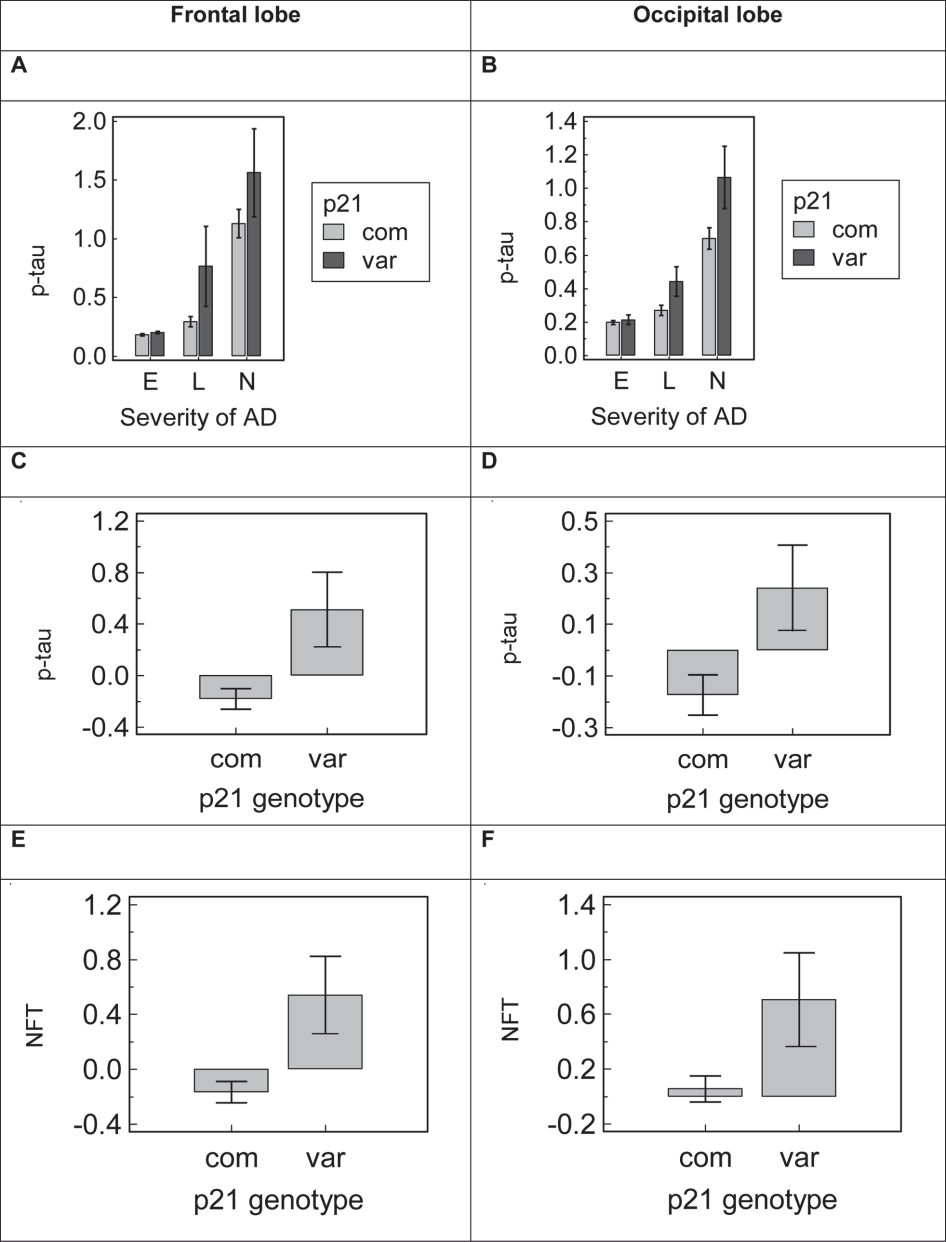
The effect of the p21^cip1^ genotype on the expression of tau pathology in the brain. **Panel A and B**: The graphs shows the mean p-tau levels in the brain of subjects in subgroups defined by the severity of AD and the p21^cip1^ genotype (Panel A: frontal lobe, Panel B: occipital lobe). The x-axis represents the severity of AD as defined by Braak: E = entorhinal stage, L = limbic stage, N = neocortical stage. The y-axis represents the amount of p-tau detected in the relevant lobe by ELISA with a marker for AT8 (arbitrary units). Light grey bars: subjects with common p21^cip1^; dark grey bars: subjects with variant p21^cip1^. Statistical test: Kruskal Wallis. **Panel C, D, E and F**: Z-scores were calculated for the amount of p-tau and NFT detected in the temporal, frontal and occipital lobe of each subject, taking into account the severity of AD as defined by Braak. This eliminated the need for subgroups defined by the disease severity. The graphs show the mean z-scores in subgroups defined by the p21^cip1^ genotype. Panel C: p-tau in the frontal lobe. Panel D: p-tau in the occipital lobe. Panel E: NFT in the frontal lobe. Panel F: NFT in the occipital lobe. The x-axes represent the p21^cip1^ genotype, with com and var representing subjects with common and variant p21^cip1^ respectively. The y-axes represents the p-tau or NFT content of the relevant brain region as determined by ELISA with markers for AT8 and DC11 respectively (arbitrary units). Statistical test: one-way ANOVA. The top/bottom of the bars represent the mean. The error bars represent the SEM.

Of the investigated pathological features, the p21^cip1^ genotype had the greatest effect on the accumulation of tau pathology in the brain. At post-mortem, subjects with variant p21^cip1^ had significantly greater amounts of hyperphosphorylated tau (p-tau) and neurofibrillary tangles (NFT) in the frontal lobe than subjects with common p21^cip1^, irrespective of the disease severity (p = 0.003 and p = 0.003 for p-tau and NFT respectively). Subjects with variant p21^cip1^ also had significantly greater p-tau and NFT accumulation in the occipital lobe than subjects with common p21^cip1^: with p-tau results reaching statistical significance in subjects with mild and advanced AD only (p = 0.014 and p = 0.029 respectively) and NFT results reaching significance irrespective of the severity of disease (p = 0.023) (Fig. 5). The p21^cip1^ genotype had no effect on the accumulation of tau pathology in the temporal lobe, irrespective of AD severity (data not shown).

**Figure 6:**
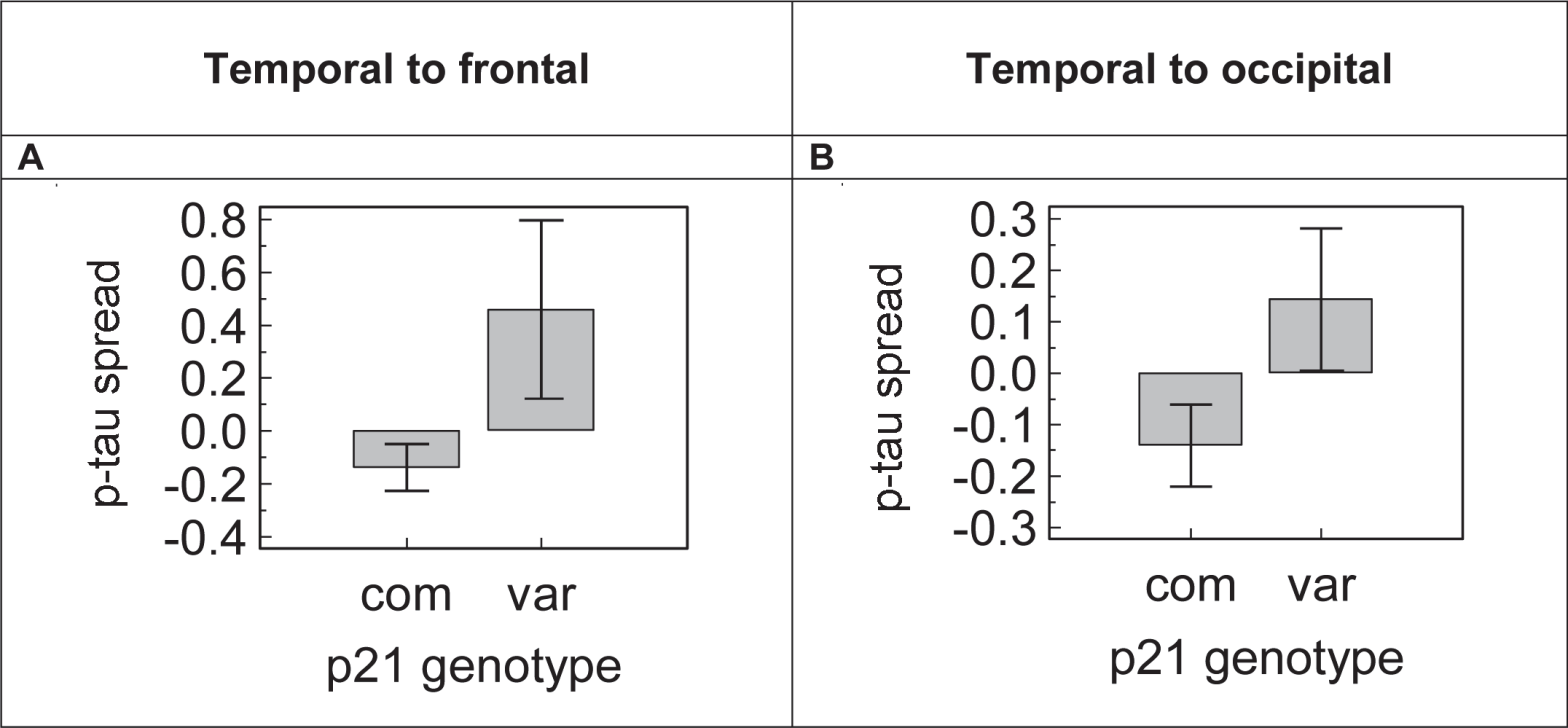
The effect of the p21^cip1^ genotype on the spread of p-tau pathology in the brain. Panel A and B. The spread of p-tau pathology was defined as the ratio of the amount of p-tau in the region less severely affected by AD (Panel A: frontal lobe; Panel B: occipital lobe) over that in the more severely affected region (temporal lobe). Z-scores were calculated for the spread of p-tau taking into account the disease severity as defined by Braak, which eliminated the need for subgroups defined by the disease severity. The graphs show the mean z-scores of p-tau spread in subgroups defined by the p21^cip1^ genotype. The x-axis represents the p21^cip1^ genotype, with com and var representing subjects with the common and variant p21^cip1^ respectively. The y-axis represents the spread of p-tau calculated from data determined by ELISA with a marker for AT8 (arbitrary units). The error bars represent the SEM. Statistical test: one-way ANOVA of z-scores.

We analysed the effect of the p21^cip1^ genotype on the spread of tau pathology from the temporal to the frontal and occipital lobe. Spread of pathology was defined as the ratio of the amount of pathology in the region less severely affected by AD over that in the more severely affected region. We found that subjects with variant p21^cip1^ had significantly greater p-tau spread from the temporal to the frontal lobe than subjects with common p21^cip1^, irrespective of the severity of disease (p = 0.025) (Fig. 6A). There was a trend (not statistically significant) for subjects with variant p21^cip1^ to have greater p-tau spread from the temporal to the occipital lobe than subjects with common p21^cip1^ (Fig. 6B). The spread of NFT from the temporal to the frontal and occipital lobe was independent of the p21^cip1^ genotype, irrespective of disease severity (data not shown).

The accumulation of beta-amyloid was independent of the p21^cip1^ genotype in all the brain regions investigated (temporal, frontal and occipital) (data not shown). Although in the frontal lobe there was a trend for the p21^cip1^ variant to be associated with lower synaptic densities and a reduction in the synaptic remodelling activity compared to the common p21^cip1^, the result did not reach statistical significance (data not shown). The synaptic density and the remodelling activity were independent of the p21^cip1^ genotype in the temporal and occipital lobe (data not shown).

**Figure 7:**
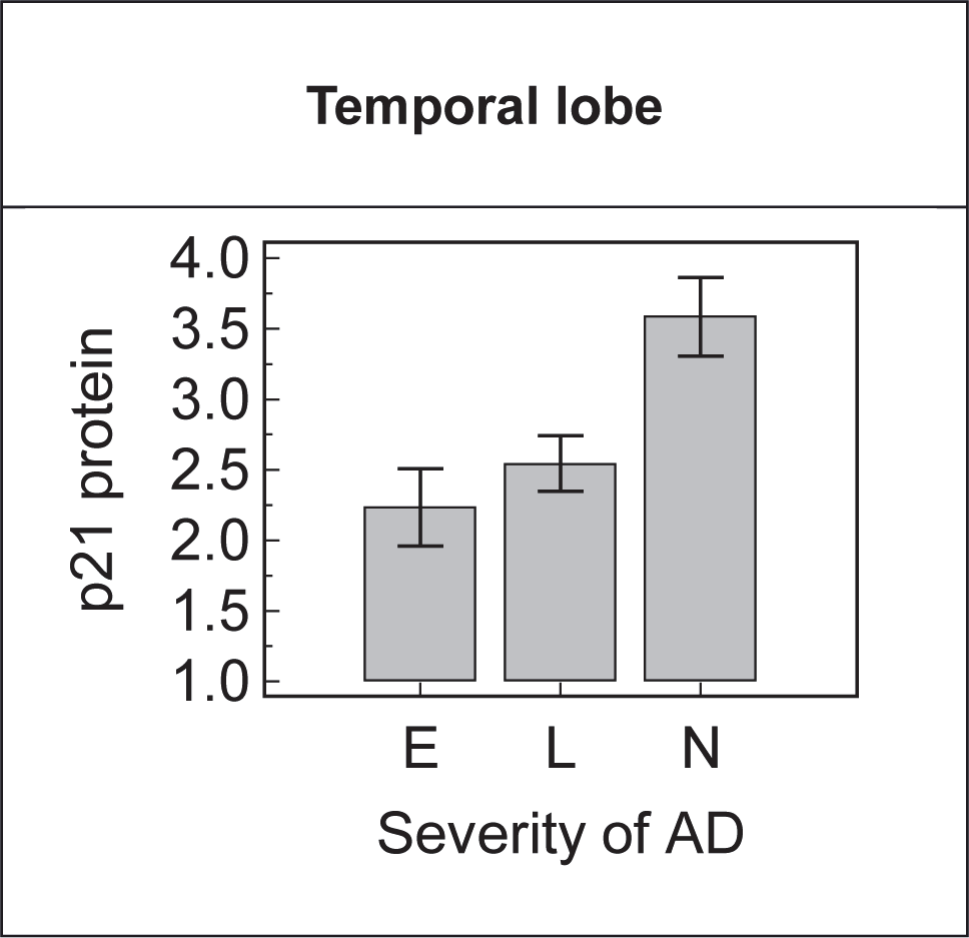
The association of the disease stage with p21^cip1^ expression in the temporal lobe. Subjects with more severe AD had a significantly greater amount of p21^cip1^ protein in the temporal lobe than subjects in an earlier stage of the disease (p-value: 0.007). The x-axis represents the severity of AD as defined by Braak: E = entorhinal stage, L = limbic stage, N = neocortical stage. The y-axis represents the p21^cip1^ protein content of the temporal lobe as determined by ELISA (arbitrary units). The top of the bars represent the mean and the error bars the SEM. Statistical test: Kruskal Wallis.

The amount of p21^cip1^ protein found in the temporal lobe, but not in the frontal and occipital lobe, was raised in subjects with more severe AD (p = 0.007) (Fig. 7). However, p21^cip1^ protein expression was independent of the p21^cip1^ genotype in all the brain regions examined (data not shown). The p21^cip1^ mRNA expression and the protein per mRNA ratio were independent of both the severity of AD and the p21^cip1^ genotype (analysed only in the frontal lobe). Multiple regression analysis showed that the expression of p21^cip1^ protein was dependent on the NFT content (R^2^ = 0.1, p = 0.003) and independent of both p21^cip1^ mRNA expression and disease severity (analysed in the frontal lobe only). Data in S3 Table.

### In-Vitro Study

In order to elucidate the effect of the p21^cip1^ SNPs on the function of the protein in-vitro, human embryonic kidney cells (HEK-293, Invitrogen), intrinsically homozygous for the common variant of p21^cip1^, were transiently transfected with a vector designed to express either the common p21^cip1^, variant p21^cip1^ (with both SNPs), or no p21^cip1^ (EV NC). P21^cip1^ content at the mRNA and protein level were determined by Q-PCR and Acumen Cytometry respectively.

Confirming the success of transfection, the p21^cip1^ transfected cells had significantly greater p21^cip1^ mRNA and protein expression than the control (EV NC); and there was no significant difference (in terms of expression) between the transfectants containing the common or variant form of p21^cip1^ (Table 7). Cells transfected with variant p21^cip1^ expressed 21.9% more p21^cip1^ protein per mRNA than cells transfected with common p21^cip1^.

**Table 7:** The ratio of p21^cip1^ protein to mRNA in the cells transfected with the different variants of p21^cip1^.

	**P21^cip1^ mRNA content (relative to control, expressed as multiple)**	**P21^cip1^ protein content (relative to control, expressed as multiple)**	**p21^cip1^ protein/ mRNA content (relative to common p21^cip1^, expressed as percentage change)**
**Common p21^cip1^**	x 298	x 7.2	N/A
**Variant p21^cip1^**	x 275	x 8.1	21.9%

The p21^cip1^ protein and mRNA results were normalised to the equivalent value for beta-actin.

**Figure 8:**
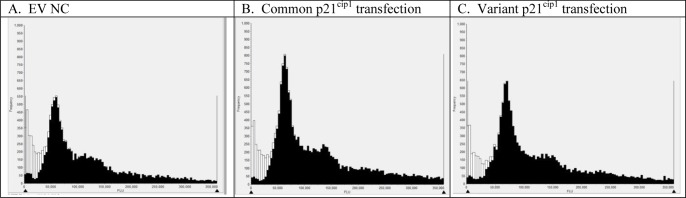
DNA distribution histograms from HEK293 cells transfected with p21^cip1^. Representative DNA distribution histograms of single cells from the Acumen cytometer (black bars). The debris and multiple cells (white bars) were excluded from the analysis. **Panel A**: Typical DNA content histogram of single cells following the transfection with the empty vector (EV NC). **Panel B**: Typical DNA content histogram of single cells following transfection with the common variant of p21^cip1^. **Panel C:** Typical DNA content histogram of single cells following transfection with the rare variant of p21^cip1^.

**Figure 9:**
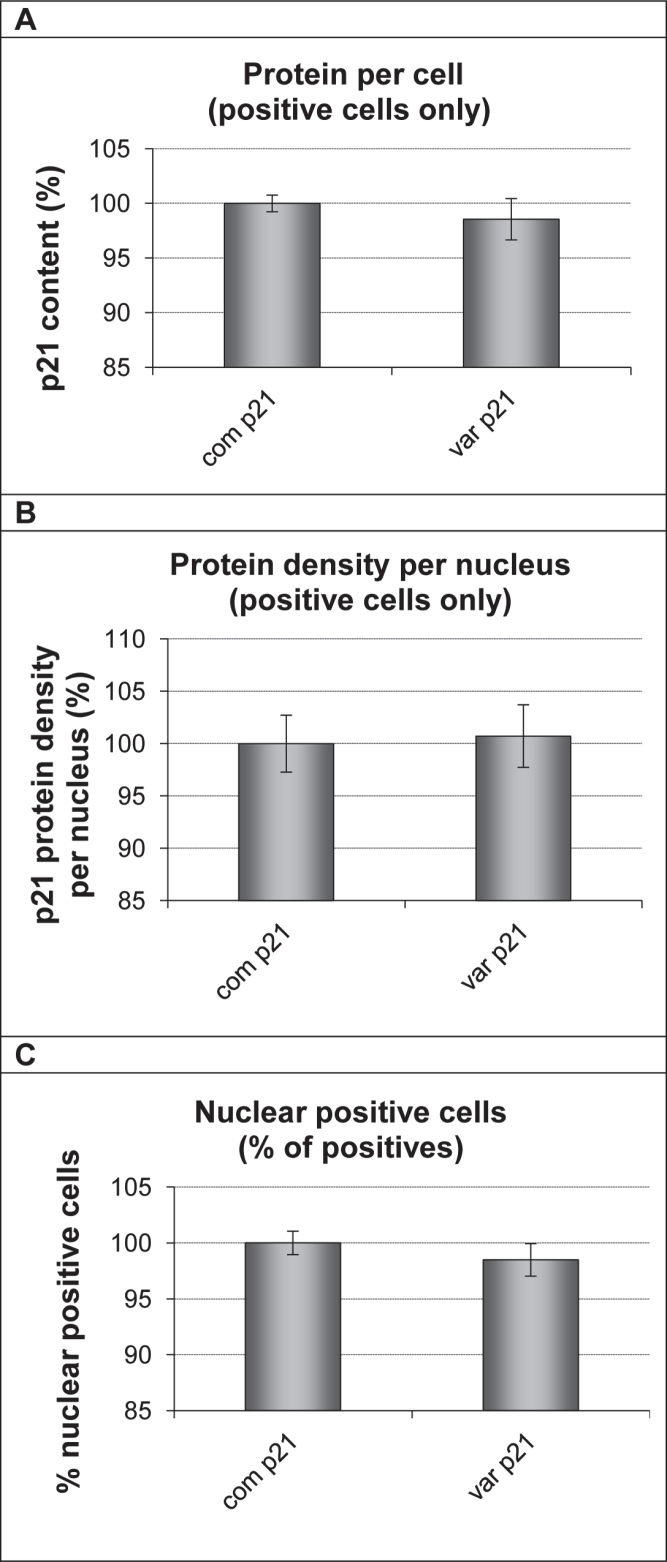
The effect of the p21^cip1^ genotype on the expression of p21^cip1^. The graphs plot the amount of p21^cip1^ protein expressed per cell (Panel A); the p21^cip1^ protein density per nucleus (Panel B) and the percentage of nuclear positive cells (Panel C) of cells transfected with either the common or variant p21^cip1^. Only cells that were positive for p21^cip1^ protein, as determined by Acumen Cytometry, were included in the analysis. To allow comparison of the cells transfected with the different variants of p21^cip1^, the expression of p21^cip1^ in each transfected population was normalised to that in the common p21^cip1^ transfected population. All results concerning p21^cip1^ expression in the common population are therefore displayed as 100%. On the x-axis: com p21 represents cells transfected with common p21^cip1^; var p21 represents cells transfected with variant p21^cip1^. The y-axis represents the p21^cip1^ protein expression per cell (Panel A), the p21^cip1^ density per nucleus (Panel B), and the percentage of nuclear positive cells (Panel C) expressed as a percentage of that in the population transfected with common p21^cip1^. The top of the bars represent the mean. The error bars represent the SEM. Analysis was carried out from 8 technical replicates of more than 5000 cells each.

Within each well the gates for the DNA histogram were set using the whole population (Fig. 8) but cells that were positive and negative for p21^cip1^ protein (as determined by Acumen Cytometry Fig. 1) were analysed separately to avoid misinterpretation of data as a result of unequal transfection efficiency or seeding density. The transfection efficiency was relatively low for both variants (12–13% p21^cip1^ positive cells after 36 hours; ~21% transfection efficiency) but the number of p21 positive cells was above 1000/well to allow meaningful analysis. The p21^cip1^ genotype had no effect on the p21^cip1^ protein expression per cell, p21^cip1^ protein density per nucleus, or on the percentage of p21^cip1^ nuclear positive cells when only p21^cip1^ positive cells were analysed (Fig. 9). This indicates that the SNPs did not alter either the expression or the nuclear translocation efficiency of the p21^cip1^ protein.

**Figure 10:**
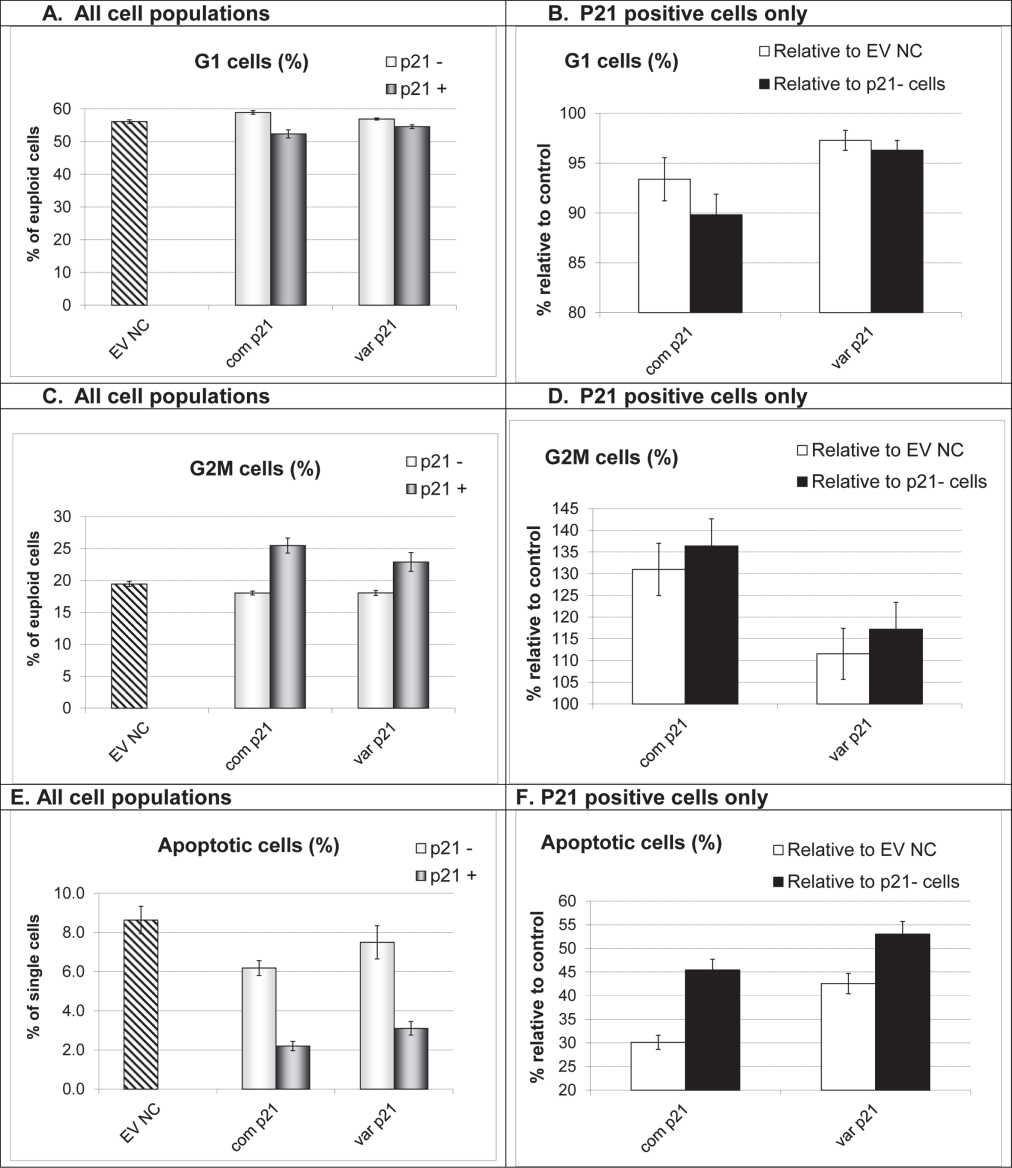
The effect of the p21^cip1^ genotype on the cell cycle and apoptotic activity. **Panel A:** The fraction of cells in the G1 phase of the cell cycle (from the euploid population) in the different cell populations. EV NC: empty vector negative control; com p21: the common p21^cip1^; var p21: variant p21^cip1^. For the cells transfected with p21^cip1^: the light grey bars represent the non-transfected cells (cells negative for p21^cip1^, p21-) and the dark grey bars represent the transfected cells (cells that were positive for p21^cip1^, p21+). The top of the bars represent the mean. The error bars represent the SEM. **Panel B:** p21 positive cells only. The fraction of cells in the G1 phase of the cell cycle normalised to control. EV NC: empty vector negative control. P21-: non-transfected cell population within the same wells. White bars: p21 positive population as a percentage of the EV NC (100% represents G1 fraction identical to that seen in EV NC cultures). Black bars: p21 positive population as percentage of the p21- population (100% represents G1 fraction identical to that seen in p21- cells). X-axis: com p21: the common p21^cip1^; var p21: variant p21^cip1^. The top of the bars represent the mean. The error bars represent the SEM. **Panel C:** The fraction of cells in the G2 phase of the cell cycle (from the euploid population) in the different cell populations. EV NC: empty vector negative control; com p21: the common p21^cip1^; var p21: variant p21^cip1^. For the cells transfected with p21^cip1^: the light grey bars represent the non-transfected cells (cells negative for p21^cip1^, p21-) and the dark grey bars represent the transfected cells (cells that were positive for p21^cip1^, p21+). The top of the bars represent the mean. The error bars represent the SEM. **Panel D:** p21 positive cells only. The fraction of cells in the G2 phase of the cell cycle normalised to control. EV NC: empty vector negative control. P21-: non-transfected cell population within the same wells. White bars: p21 positive population as a percentage of the EV NC (100% represents G2 fraction identical to that seen in EV NC cultures). Black bars: p21 positive population as percentage of the p21- population (100% represents G2 fraction identical to that seen in p21- cells). X-axis: com p21: the common p21^cip1^; var p21: variant p21^cip1^. The top of the bars represent the mean. The error bars represent the SEM. **Panel E:** The fraction of apoptotic cells (from single cell population) in the different cell populations. EV NC: empty vector negative control; com p21: the common p21^cip1^; var p21: variant p21^cip1^. For the cells transfected with p21^cip1^: the light grey bars represent the non-transfected cells (cells negative for p21^cip1^, p21-) and the dark grey bars represent the transfected cells (cells that were positive for p21^cip1^, p21+). The top of the bars represent the mean. The error bars represent the SEM. **Panel F:** p21 positive cells only. The fraction of apoptotic cells normalised to control. EV NC: empty vector negative control. P21-: non-transfected cell population within the same wells. White bars: p21 positive population as a percentage of the EV NC (100% represents apoptotic fraction identical to that seen in EV NC cultures). Black bars: p21 positive population as percentage of the p21- population (100% represents apoptotic fraction identical to that seen in p21- cells). X-axis: com p21: the common p21^cip1^; var p21: variant p21^cip1^. The top of the bars represent the mean. The error bars represent the SEM. Analysis was carried out from 8 technical replicates of more than 5000 cells each.

Despite comparable expression and nuclear translocation efficiency, the effect of p21^cip1^ on the cell cycle kinetics and apoptotic activity was dependent on the p21^cip1^ genotype. The increase in the G2 population (G2 phase arrest) was significantly stronger with the common p21^cip1^ (41% relative to non-transfected cells and 31% relative to the EV NC) compared to variant p21^cip1^ (20% relative to non-transfected cells and 12% relative to the EV NC). Furthermore, the reduction in the rate of apoptosis was significantly greater with the common p21^cip1^ (42% relative to non-transfected cells and 30% relative to the EV NC) compared to variant p21^cip1^ (49% relative to non-transfected cells and 43% relative to the EV NC) (Fig. 10, Data in S4 Table).

**Figure 11:**
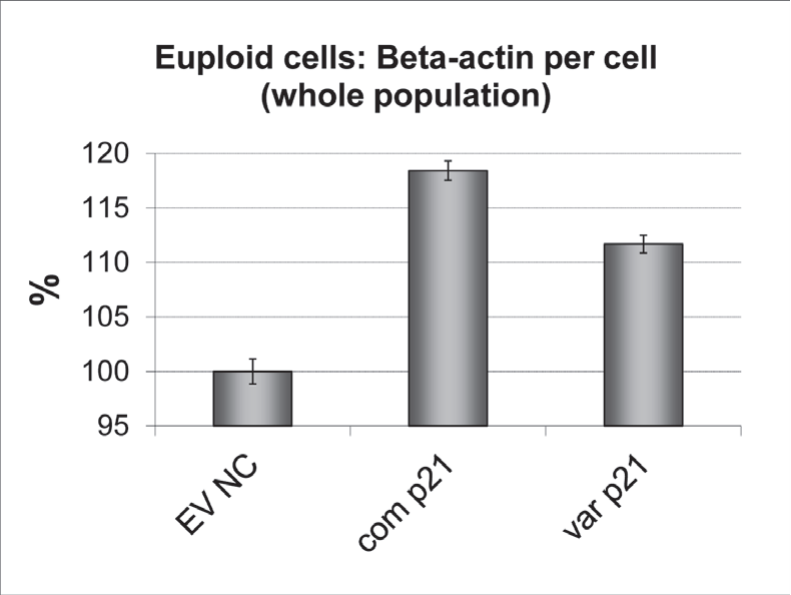
The effect of the p21^cip1^ genotype on beta-actin expression. The graph shows the amount of beta-actin detected per cell expressed as a percentage change relative to the empty vector negative control. Beta-actin expression was determined by Acumen Cytometry with beta-actin immunostaining: with the total amount of beta-actin detected divided by the total number of cells in the sample. The cells were not double stained for p21^cip1^ protein, so we were unable to differentiate the p21^cip1^ positive population (transfected cells) from the p21^cip1^ negative population (non-transfected cells). X-axis: EV NC: Empty vector negative control; com p21: cells transfected with common p21^cip1^; var p21: cells transfected with variant p21^cip1^. The y-axis represents the percentage change in beta-actin expression per cell relative to the EV NC. The top of the bars represent the mean. The error bars represent the SEM.

Whilst both forms of p21^cip1^ increased the beta-actin expression per cell, the size of the increase was dependent on the genotype. Cells transfected with common p21^cip1^ expressed a significantly greater amount of beta-actin per cell (~18% more than the EV NC) than cells transfected with variant p21^cip1^ (~11% more than the EV NC) (Fig. 11). The p21^cip1^ expressing cells were also significantly larger than their non-transfected counterparts. However, the presence of the SNPs did not alter this p21^cip1^-dependent increase in cell size (data not shown).

## Discussion

The CDK inhibitor: p21^cip1^ is capable of inducing cell cycle arrest at the G1/S and G2/M checkpoints [59]. It is up-regulated on a transcriptional level in response to p53 (a DNA-damage induced transcription factor), and inhibits CDK2/cyclin A and CDK2/ cyclin E, thereby acting as a G1/S checkpoint inhibitor. Additionally, a number of studies indicate that p21^cip1^, expressed independently of p53, induces cell cycle arrest at the G2/M checkpoint [28–31]. P21^cip1^ has a number of additional cellular functions, such as DNA replication and repair [60] and the prevention of apoptosis [61]. The subcellular localisation of p21^cip1^ may dictate its function: with a nuclear location required for its cell cycle regulatory role, and a cytoplasmic location required for its anti-apoptotic role [62,63]. The p21^cip1^ protein has specific binding sites for a large number of different proteins, including cyclins, CDKs, PCNA, ASK1, procaspase 3, SAPK, TSG101, E2F-1, and WISp39 [33].

We hypothesised that two cancer associated SNPs of p21^cip1^ may reduce p21^cip1^ protein expression or function, thereby contributing to the loss of G1/S checkpoint control in neurons that is postulated to be a cause of neurodegeneration in AD. Our results confirmed that p53-independent expression of p21^cip1^ leads to G2/M checkpoint inhibition and protection against apoptosis. We found that the SNPs, although they did not affect expression or nuclear translocation, reduced both the cell cycle inhibitory and anti-apoptotic activity of p21^cip1^.

Additionally, the SNPs did not alter the p21^cip1^-dependent increase in cell size, but reduced the p21^cip1^- dependent increase in cellular beta-actin expression. Beta-actin is an integral component of the cellular cytoskeleton, which is extensively re-organised during the cell cycle and is upregulated during cellular differentiation [64,65]. The effect of the SNPs on the up-regulation of beta-actin suggests that the SNPs reduced the ability of p21^cip1^ to induce differentiation. Furthermore, the p21^cip1^ SNPs altered the p21^cip1^ protein to mRNA ratio: with a significantly greater amount of p21^cip1^ protein found per mRNA for variant p21^cip1^ than for the common p21^cip1^. This suggests that the SNPs either reduced the stability of the p21^cip1^ mRNA or reduced the rate of degradation of the p21^cip1^ protein.

Whilst we cannot draw conclusions on the individual effects of the p21^cip1^ SNPs, it is known that the SNP at codon 31 of p21^cip1^ induces an amino acid change within the binding domain of WISp39, ASK1, procaspase 3, SAPK, TSG101 and E2F-1 [33]. WISp39 increases the stability of the p21^cip1^ protein; ASK1, procaspase 3 and SAPK are pro-apoptotic proteins that are inhibited by p21^cip1^; TSG101 increases the stability of p21^cip1^/cyclin/CDK2 complexes, thereby enhancing the cell cycle inhibitory effects of p21^cip1^; whereas E2F-1 promotes E2F-dependent transcription of cell cycle promoting proteins and is inhibited by p21^cip1^ binding. The SNP is likely responsible for a loss of strength of binding of p21^cip1^ to some or all of these proteins. The reduction in the cell cycle inhibitory activity induced by the SNPs may be due to a loss of strength of binding of p21^cip1^ to TSG101 and E2F-1, whereas the reduction in the anti-apoptotic activity may be due to a loss of strength of binding to the pro-apoptotic proteins (ASK1, procaspase 3 and SAPK). In addition, the SNP may alter the affinity of the p21^cip1^ protein for its binding partners, by altering its preferred tertiary structure. Whilst the p21^cip1^ protein is an intrinsically unstructured protein [36], its preference for certain conformational arrangements will depend on the charges of its residues. The serine (polar uncharged amino acid) to arginine (positively charged amino acid) change induced by the SNP at codon 31 may lead to a change in the preference of the p21^cip1^ protein for its binding partners.

Although the SNP at codon 31 lies within the binding domain of WISp39 (a protein that stabilises p21^cip1^), we found that the SNP was associated with a greater protein per mRNA ratio. Further studies are necessary to elucidate whether this effect is due to altered protein or mRNA stability [32].

The requirement for co-localisation of the p21^cip1^ SNPs for an association with cancer [19,21] suggests that the individual effects of the SNPs are required together for a significant loss of G1/S checkpoint control. We have shown that the SNPs, when together, reduce the function of the p21^cip1^ protein and may alter the stability of it. All our findings were consistent with the known association of the p21^cip1^ SNPs with cancer in populations of European descent [16–22]: the population group where the SNPs are the least prevalent [66]. The relatively small effect of the variant on the p21^cip1^ function, together with the possible compensatory effect of other members of the kip/cip family, may explain the low-penetration of this risk factor for cancer [20]. The lack of association between these SNPs and cancer in other ethnic groups [20] may be due to other genetic and environmental factors that may either impact on the functional effects of the SNPs or affect the development of cancer.

Since there are no adequate cellular models to study the effect of p21^cip1^ SNPs on AD-type protein expression, we analysed these relationships in a series of post mortem brain samples from AD patients and controls. In the post-mortem study, we found that p21^cip1^ levels were raised in brain regions affected by AD. The expression of p21^cip1^ protein was most strongly dependent on the accumulation of tangle pathology, and was independent of the p21^cip1^ genotype or p21^cip1^ mRNA levels. The fact that p21^cip1^ protein expression was only related to tangles, but not to the p21^cip1^ mRNA or to the amyloid component of the pathology, would suggest that the p21^cip1^ protein may bind non-specifically to tangles as previously described [39,67,68]. Therefore, in post-mortem studies, the raised expression of p21^cip1^ protein in the AD brain may be an artefact due to inappropriate accumulation, rather than a cause of AD-type protein expression as suggested by in vitro studies [53]. On the other hand, the finding of p21^cip1^ overexpression in peripheral lymphoblasts of AD patients does warrant further investigations into the role of the protein in the disease process [69,70].

Confirming the result of a previous study [23], we found an association between the p21^cip1^ variant and AD. The variant was associated with a relatively small but significantly increased risk of AD.

Although the variant was associated with an increased risk of PD with dementia relative to age matched controls, it was not associated with dementia in PD relative to non-demented PD patients. The latter could be the result of the relatively small patient numbers in the two groups. Since dementia is mostly attributed to the appearance of AD-type pathology in PD patients [71,72], it is not surprising that the relative frequency of the variant allele and the associated odds ratios are very similar in the AD and PD with dementia groups. Prior to the age of 75, the variant was associated with a reduction in the dementia free survival, in relation to both AD, and PD with dementia. This effect disappeared above the age of 75. Age is the strongest risk factor for both AD and dementia associated with PD. The data indicates that this risk is increased by the relatively weak effect of the p21^cip^ variant in younger people by bringing the age of onset forward. However, in older patients (above the age of 75) the general effect of age predominates and the effect of the p21^cip1^ variant disappears.

Additionally, the SNPs were associated with an earlier age of onset of dementia in AD, and a more severe AD phenotype, with a primary influence on the accumulation of tangle pathology in brain regions that are affected relatively late in the disease process. This, and the lack of a similar relationship between the SNPs and temporal lobe pathology, may be a reflection of the fact that the tau accumulation in the temporal lobe may have reached a ceiling in the patients, whilst the formation of tau-related pathology in the frontal and occipital regions was still ongoing. The genotype-phenotype correlation also suggests that the p21^cip1^ SNPs have a direct effect on p-tau accumulation and NFT formation consistent with the involvement of cell cycle deregulation in the pathogenesis of AD. The finding is also consistent with recent findings of deficient p53-dependent DNA damage repair, which requires a fully functional p21^cip1^ [59] in AD [73]. However, we found no relationship between the p21^cip1^ SNPs and amyloid accumulation in the same brain regions. This may be partly due to the fact that beta-amyloid accumulation tends to reach a plateau early in the disease process. Alternatively, the complex relationship between DNA-damage-repair signalling, apoptosis and beta amyloid [74] may be dependent on other risk factors (genetic and environmental) that either have a stronger effect on beta-amyloid accumulation than these SNPs or alter the effect of them.

Historically, the ApoE4 risk factor has been associated with a significantly more severe AD-type pathology in post mortem series [75,76]. A relatively recent review found that several other identified genetic risk factors for AD show no similar genotype-phenotype relationship [77] suggesting that these SNPs, whilst affecting AD susceptibility, do not have a direct effect on tau and amyloid accumulation. The relationship between an earlier age of onset and more severe tangle pathology and the functionally defective p21^cip1^ variant supports the hypothesis that the p21^cip1^ variant, by contributing to the loss of cell cycle control in neurons, is directly involved in the pathogenesis of AD [38,39].

## Supporting Information

S1 Table Demographic data on the patient groups included in the genotyping study. CONTROL: healthy elderly individual; AD: Alzheimer’s disease; OD: Other dementia syndromes; AD/PD: Mixed Alzheimer’s disease and Parkinson’s disease; MCI: Mild cognitive impairment; PD: Parkinson’s disease; PD D: Parkinson’s disease with dementia.
(XLS)

S2 Table Demographic data of patients with PM data. Pre-clinical: Entorhinal stage Alzheimer’s disease (Braak I and II); Mild AD: Limbic stage Alzheimer’s disease (Braak III and IV); Advanced AD: Neocortical stage Alzheimer’s disease (Braak V and VI).
(XLS)

S3 Table Data in PM study. Pre-clinical: Entorhinal stage Alzheimer’s disease (Braak I and II); Mild AD: Limbic stage Alzheimer’s disease (Braak III and IV); Advanced AD: Neocortical stage Alzheimer’s disease (Braak V and VI). Protein measures (p-tau and p21) are arbitrary units from ELISA assay. SEM: Standard Error of the Mean.
(XLS)

S4 Table Different cell populations as measured by flow cytometry. EV NC: empty vector negative control; com p21: the common p21^cip1^; var p21: variant p21^cip1^; p21-: non-transfected cells (cells negative for p21^cip1^); p21+: transfected cells (cells that were positive for p21^cip1^).
(XLS)
